# TB-related catastrophic costs in Ethiopia

**DOI:** 10.5588/pha.24.0006

**Published:** 2024-06-01

**Authors:** A.A. Deribew, Z.G. Dememew, K.M. Alemu, G. Tefera, S.G. Negash, Y.A. Molla, A.G. Woldegiorgis, D.G. Datiko, P.G. Suarez

**Affiliations:** ^1^ United States Agency for International Development (USAID) Eliminate TB Project, Management Sciences for Health (MSH), Addis Ababa,; 2 USAID Eliminate TB Project, KNCV Tuberculosis Foundation, Addis Ababa, Ethiopia;; 3 MSH, Arlington, VA, USA

**Keywords:** tuberculosis, Ethiopia, catastrophic cost

## Abstract

**OBJECTIVES:**

To measure the progress towards reducing TB-related catastrophic costs in 19 zones of Amhara, Oromia, SNNP (Southern Nations and Nationalities, and Peoples) and Sidama Regions of Ethiopia.

**METHODS:**

A baseline survey was conducted in randomly selected health facilities from all districts within the 19 zones from November 2020 to February 2021. Interventions targeting the major drivers of catastrophic costs identified in the baseline survey, such as installation of 126 GeneXpert and 13 Truenat machines, securing connectivity of 372 GeneXpert, establishing alternative specimen referral systems, and capacity-building of health workers, were implemented. A follow-up survey was conducted from October to December 2022. The WHO generic tool was used to collect data based on probability proportional to size. Data were entered into STATA software, and the proportion of catastrophic costs was calculated and compared between the two surveys.

**RESULTS:**

A total of 433 and 397 patients participated in the baseline and follow-up surveys, respectively. The proportion of catastrophic costs reduced from 64.7% to 43.8% (*P* < 0.0001). The share of direct non-medical costs decreased from 76.2% to 19.2%, while medical and indirect costs increased from 11.6% and 12.3% to 30.4% and 52.4 %.

**CONCLUSION:**

The proportion of households facing TB-related catastrophic costs has significantly reduced over the 2-year period. However, it remains unacceptably high and varies among regions. Further reducing the catastrophic costs requires multisectoral response, reviewing the TB service exemption policy, further decentralisation and improving the quality of TB services.

## Abstract (fr)

**OBJECTIFS:**

Mesurer les progrès accomplis dans la réduction des coûts catastrophiques liés à la TB dans 19 zones des régions d'Amhara, d'Oromia, de SNNP (Région des nations, nationalités et peuples du Sud) et de Sidama en Éthiopie.

**MÉTHODES:**

Une enquête de base a été menée dans des établissements de santé sélectionnés au hasard dans tous les districts des 19 zones de novembre 2020 à février 2021. Des interventions ciblant les principaux facteurs de coûts catastrophiques identifiés dans l'enquête de référence, telles que l'installation de 126 machines GeneXpert et 13 Truenat, la sécurisation de la connectivité de 372 GeneXpert, la mise en place de systèmes alternatifs d'orientation des échantillons et le renforcement des capacités des agents de santé, ont été mises en œuvre. Une enquête de suivi a été menée d'octobre à décembre 2022. L'outil générique de l'OMS a été utilisé pour recueillir des données fondées sur une probabilité proportionnelle à la taille. Les données ont été saisies dans le logiciel STATA, et la proportion des coûts catastrophiques a été calculée et comparée entre les deux enquêtes.

**RÉSULTATS:**

Au total, 433 et 397 patients ont participé respectivement à l'enquête de base et à l'enquête de suivi. La proportion des coûts catastrophiques est passée de 64,7% à 43,8% (*P* < 0,0001). La part des coûts non médicaux directs a diminué, passant de 76,2% à 19,2%, tandis que les coûts médicaux et indirects sont passés de 11,6% et 12,3% à 30,4% et 52,4%.

**CONCLUSION:**

La proportion de ménages confrontés à des coûts catastrophiques liés à la tuberculose a considérablement diminué au cours de la période de 2 ans. Cependant, il reste inacceptable et varie selon les régions. Pour réduire davantage les coûts catastrophiques, il faut une réponse multisectorielle, une révision de la politique d'exemption des services de lutte contre la TB, une décentralisation plus poussée et une amélioration de la qualité des services de lutte contre la TB.

TB is one of the top infectious disease killers worldwide, and the emergence of multidrug-resistant TB (MDR-TB) and extensively drug-resistant TB (XDR-TB) poses a significant challenge to global health. TB causes significant productivity losses due to illness, disability, and premature death, leading to economic hardship for patients and their families.^1^

Particularly in low-income settings where access to affordable healthcare is limited, the costs associated with diagnosis and treatment of TB force many households to cut down on necessities such as food, clothing, and children’s education or they decide not to use services simply because they cannot afford them, which in turn contribute to poor health outcomes and increase the risk of disease transmission.^2,3^ Particularly, the poor and vulnerable segments of society are pushed into a vicious cycle of ill health and poverty.^4^

The WHO report indicates that, globally, approximately 50% of TB patients and their families faced catastrophic costs in 2022, defined as direct medical expenditures, direct non-medical expenditures, and indirect costs (e.g., income losses) that total more than 20% of household income.^5^ The average income loss due to TB illness can be equivalent to more than one year’s income,^6^ and even more among MDR-TB patients and their households.^7^

Ethiopia has made significant progress in extending healthcare services to rural and disadvantaged urban areas, with over 94% of the population now having access to primary healthcare.^8^ TB diagnosis and treatment has been integrated within the primary healthcare settings to enable early detection and prompt initiation of treatment and is offered free of charge at all public health facilities.^9^ TB confirmatory diagnosis services such as Xpert^®^ MTB/RIF (Cepheid, Sunnyvale, CA, USA) and X-rays for clinical diagnosis have been expanded to diagnostics in more than 350 hospitals and high TB burden health centres, while advanced diagnosis such as liquid and solid culture and whole genome sequencing are also available at regional and zonal levels. Community-based TB care, engaging community health workers and volunteers, has also been initiated to enhance TB case identification, treatment adherence, and monitoring, especially in remote and underserved areas.

Consequently, the incidence of TB has decreased from 421/100,000 people in 2000 to 119/100,000 people in 2022. Despite this significant decline, Ethiopia remains among the 30 countries with a high burden of TB, with over 145,000 people contracting the disease and more than 19,000 deaths attributed to TB. Additionally, the WHO estimates that approximately 30% of TB cases go undetected by the healthcare system.^10^ While a national study on the TB catastrophic cost survey has not been conducted, a smaller-scale study focusing solely on direct costs and another meta-analysis study suggested that 40% and 51% of TB patients face catastrophic costs.^11,12^ These studies also showed that private facility diagnosis, drug-resistant TB, TB-HIV co-infection, hospitalisation, household income, and diagnosis delays were the major factors associated with catastrophic costs.^11–13^

Ethiopia has adopted the WHO End TB strategy,^14^ which achieves ‘Zero TB-related catastrophic costs’ by 2025.^15^ The Management Sciences for Health, through the USAID Eliminate TB Project, provides support for national TB control efforts in Amhara, Oromia, Southern Nations, Nationalities, and Peoples (SNNP), and Sidama Regions. Therefore, this study seeks to assess the advancement in reducing catastrophic costs within the framework of the project, serving as valuable information for policymakers at all levels to make evidence-based decisions.

## METHODS

### Study setting and design

We conducted health facility-based cross-sectional TB cost surveys at two different points in 19 zones of Amhara, Oromia, SNNP, and Sidama Regions of Ethiopia, where the project is functioning. Based on projections from the 2007 Census, these zones constitute an estimated 42,806,707 people in 2022,^16^ and more than 1,050 public health centres, 58 hospitals, and 26 private health facilities provide TB diagnosis and treatment services. The is health centres serve as the first point of contact for TB diagnosis and treatment, where they collect samples from presumptive cases, transport them to the nearest diagnostic sites, or refer patients for clinical diagnosis. They also initiate DOTS for confirmed TB cases, provide adherence support, and monitor treatment outcomes using sputum smear microscopy. Assuming a TB incidence rate of 132 cases per 100,000 population and a case detection rate of 70%, during the baseline survey, we anticipate approximately 19,638 TB patients per 6 months. distributed among the health facilities, and 2–4 patients visit the health facility for follow-up or medication collection each month.

As part of the project, we conducted a baseline survey from November 2020 to February 2021 to understand the extent and factors associated with TB-related catastrophic costs. After implementing various interventions, a follow-up survey was conducted from October to December 2022.

### Intervention

Various interventions targeting the major drivers of catastrophic cost and improving access to diagnostic and treatment services have been implemented. This included installing an additional 126 GeneXpert machines and 13 Truenat (Molbio Diagnostics, Verna, India) machines and ensuring the connectivity of 372 GeneXpert machines. In addition, an alternative specimen referral system was established that enabled remote health facilities to transport TB specimens to diagnostic centres using non-healthcare workers, thereby ensuring continuous service while healthcare workers focus on their duties. In addition, enhanced support was provided to further decentralise TB DOTS services to health posts and the community level, particularly for those in remote areas. This was crucial for reducing the time and financial costs of long-distance travel and improving treatment outcomes.

Furthermore, several capacity-building training sessions and supportive supervision programmes were conducted to improve the quality of TB diagnosis and treatment and ensure a consistent supply of drugs and supplies. Overall, these interventions not only enhance access to and quality of TB diagnosis and treatment services but also reduce the need for long-distance travel and multiple healthcare visits before diagnosis. They also reduce the time and expenses associated with patient referrals for diagnosis and travel to health centres for DOT services.

### Sampling method

The sample size was determined using the single point calculation formula, 41% catastrophic costs,^2^ and an absolute precision of 4%. This yielded, with an additional 10 % contingency, a sample of 460 patients and 424 patients in the baseline and follow-up surveys, respectively. Consequently, one-third of the health facilities providing DOTs were selected from each district using the rule of thumb, and the estimated sample size was distributed proportionally across all health facilities.

The data collection involved customising the WHO generic TB cost survey,^1^ which was previously used in Vietnam^17^ and China,^18^ adopted to local language and context. The project staff, who were also trained on the questionnaire, data collection approach, and ethical considerations, collected the data via a paper-based questionnaire while patients visited the health facility for follow-up or to collect their drugs. The average household annual income and direct and indirect costs incurred (operational definition in Table 1) in the intensive and continuous phases of treatment were collected on the basis of patient response.

**TABLE 1.** Definitions of indirect, direct, and catastrophic costs.

Type of cost	Elements included in cost type	Methods used to calculate costs
Direct medical costs	Cost of consultations, laboratory tests, and other medical procedures	Sum of all medical costs net of any reimbursements
Direct non-medical costs	Transportation costs Cost of accommodations, food, nutritional supplements Hourly wage lost due to time loss, visiting health facilities for diagnosis and DOT, or a visit to pick up TB drugs	Sum of direct non-medical and direct medical costs net of any reimbursements
Indirect costs	Lost productivity or wage due to illness	Sum of the reported product or wages during the TB episode, as reported by an individual

DOT = directly observed therapy.

### Data management, validation, and analysis

The data were entered into STATA software (Stata, College Station, TX, USA) by clerks who received a 1-day orientation, and double entry was performed for every twentieth questionnaire to ensure data quality. Based on the WHO protocol,^19^ we calculated the total medical costs, total travel costs, total food costs, total accommodation costs, and total income lost for the particular episode phase the patient is in, and the cost for other phases was estimated from similar patients (matched by type of TB and facility) interviewed in the other phases of illness. Then, we calculated the total cost of each patient during the DOTS period, and if the total costs, net of transfers and reimbursements, exceeded 20% of the total annual household’s pre-TB income, they were considered catastrophic. We used a binary response model to identify the factors most significantly associated with catastrophic costs, using a χ^2^ test with two-tailed *P* < 0.05 considered to be the threshold for statistical significance. In addition, the proportion and mean difference of the direct and indirect costs in the two surveys were compared, and *P* < 0.05 were used to determine the level of significance difference.

### Ethical considerations

We obtained support letters and permission from each study region’s ethical review committee. Oral consent was obtained before the interview of patients with TB. Consent was obtained from the parent or guardian of children under 14 years of age.

## RESULTS

Data were collected from 433 (94.1%) TB patients from 342 health facilities in the baseline survey and from 397 (90.2%) TB patients in 300 health facilities in the follow-up survey. Despite the slight difference in the number of patients with TB between the two surveys, the demographic and socioeconomic characteristics of the study participants were similar (Table 2). The average annual income of patients with TB in the baseline and follow-up surveys was US$715.50 and US$690.10, respectively. The number of patients enrolled from public hospitals was 74 (16.8%) during the baseline survey and 93 (23.4%) in the follow-up survey (*Z*-score 2.4, *P* = 0.02; Table 2).

**TABLE 2.** Summary of demographic and socioeconomic characteristics of TB patients at baseline and follow-up surveys.

Variables	​	TB cost survey		
	​	Baseline survey (*n =* 433)	Follow-up survey (*n =* 397)	*P*-value
	​	*n* (%)	*n* (%)	
Regions	Oromia	159 (36.8)	161 (40.6)	0.52
	Amhara	107 (24.8)	87 (21.9)	
	Sidama	24 (5.3)	40 (10.1)	
	SNNP	143 (33.1)	109 (27.5)	
Sex	Female	192 (44.3)	171 (43.0)	0.43
	Male	241 (55.7)	226 (57.0)	
Age, years	<15	15 (3.5)	16 (4.1)	0.73
	15–24	111 (25.6)	101 (25.5)	
	25–49	239 (55.2)	225 (56.7)	
	>49	68 (15.7)	54 (13.7)	
Educational status	Illiterate	138 (31.9)	130 (32.8)	0.62
	Grade 1–12	257 (59.4)	231 (58.3)	
	College/university	38 (8.8)	36 (9.0)	
Treating health facilities	Private facility	14 (3.2)	6 (1.5)	0.08
	Public health centre	345 (80.0)	298 (75.1)	0.08
	Public hospital	74 (16.8)	93 (23.4)	0.02
Treatment phase	Intensive	121 (30.0)	134 (33.8)	0.72
	Continuation	312 (70.0)	263 (66.2)	

SNNP = Southern Nations, Nationalities, and Peoples.

### Costs faced by patients with TB during baseline and follow-up surveys

The mean medical and non-medical costs were US$117.90 and US$213.00 at baseline and declined to US$41.80 and US$50.40 at follow-up (*P* < 0.0001). The mean indirect cost slightly increased from US$41.20 at baseline to US$45.70 at follow-up; however, this increase was not statistically significant (Table 3).

**TABLE 3.** Mean medical, non-medical, and indirect costs of the two surveys (US$).

Type of cost	Baseline	Follow-up	*Z*-score	*P*-value
Average annual household income	715.5	690.1	4.63	0.732
Mean medical cost	117.9	41.8	4.86	< 0.0001
Mean non-medical cost	213.0	50.4	8.38	< 0.0001
Mean indirect cost	41.2	45.7	1.83	0.76
Mean total cost	372.1	137.8	7.10	<0.001

*Exchange rate of US$1 = ETB44.3 for the baseline and US$1 = ETB52.8 for the follow-up survey. ETB = Ethiopian birr.

### Proportion of the catastrophic cost

In the baseline survey, 64.7% of TB patients faced catastrophic costs, which decreased to 43.8% in the follow-up surveys (*P* < 0.001). Significant reduction in the proportion of households experiencing TB-related catastrophic costs was observed in the Amhara, Oromia, and SNNP Regions (*P* < 0.05), but no change was observed in Sidama Region (Table 4). The share of direct non-medical cost reduced by 57.6 percentage points (from 76.2% to 18.6%) between the two surveys, while the share of direct medical cost and indirect costs increased by 18.4 and 39.2 percentage points (Table 5).

**TABLE 4.** Proportion of patients who incurred TB-related catastrophic costs in the baseline survey and the follow-up survey, by region.

Region	Participants		Catastrophic cost		Difference, %	
	Baseline *n*	Follow-up *n*	Baseline *n* (%)	Follow-up *n* (%)	*Z*-score	*P*-value
Amhara	107	87	89 (83.2)	51 (58.6)	3.8	<0.0001
Oromia	159	161	108 (67.9)	65 (40.4)	4.9	<0.0001
Sidama	24	40	14 (58.3)	20 (50.0)	1.2	0.26
SNNP	143	109	69 (48.3)	38 (34.8)	1.3	0.02
Total	433	397	280 (64.7)	174 (43.8)	6.2	<0.000 1

SNNP = Southern Nations, Nationalities, and Peoples.

**TABLE 5.** Proportion of the different costs categories in the two TB cost surveys.

Type of cost	Baseline %	Follow-up %	*Z*-score	*P*-value
Direct medical cost	11.6	30.0	8.6	<0.0001
Direct non-medical cost	76.2	18.6	21.8	<0.0001
Indirect cost	12.2	51.4	16.0	<0.0001

## DISCUSSION

The proportion of households experiencing TB-related catastrophic costs showed a 21.0 % point reduction in the follow-up surveys conducted after 2 years of implementing the interventions. This is expected given the wide range of interventions implemented following the baseline survey in the regions. Despite this encouraging achievement, the current proportion of catastrophic costs (44%) in the four regions is unacceptably high. This implies addressing the determinants of catastrophic cost; therefore, achieving the national target ‘zero catastrophic cost by 2025’ might require a commitment of high-level leadership and coordinated multisectoral actions.

The major reduction was related to direct non-medical costs, including transport and food and drink costs. This could be mainly associated with the additional TB diagnostic equipment allocated in health facilities nearest to the community, improvement of the sample transportation system by non-health workers, rather than patient referral for diagnosis, as well as decentralisation of TB treatment follow-up to health posts—all of which reduce the frequency and distance to visit health facilities.

In contrast, while the mean cost shows a slight reduction, the share of medical and indirect costs, out of the total cost, shows an increase in the follow-up survey. This could be due to the significant reduction in direct non-medical costs. However, inflation in the costs of transport, accommodation, and food items, which we did not consider in the calculation, could also contribute to the difference. It also indicates that patients are still visiting many health facilities before diagnosis and continue to experience delays, implying the need to improve the quality of TB diagnosis and treatment.

However, given the interventions implemented, continuing high out-of-pocket (OOP) spending to receive medical care is concerning. This is mainly because of the policy that exempts ‘TB diagnosis and treatment’ only, but the costs incurred before diagnosis, when patients must visit different health institutes before TB disease confirmation,^20^ diagnosis of extrapulmonary TB, and diagnosis and treatment for illness other than TB, developed due to TB disease or side effects of TB drugs, are not covered by the exemption policy.^21^

The proportion of catastrophic events has shown a significant reduction in all regions, except in Sidama Region. However, there remains a wide difference among regions, despite the same health system and policy. The Amhara Region has higher TB-related catastrophic costs. This could be due to the higher proportion of extrapulmonary TB (EPTB) cases in the region.^3^ EPTB is known to have a delay in diagnosis^4^ and is not exempted in Ethiopia,^21^ which contributes to higher OOPs.

### Limitations

The two TB catastrophic surveys in Ethiopia were undertaken when the country was in two different contexts, and factors such as the COVID-19 epidemic and the conflict in northern Ethiopia and the Oromia Region could affect the estimation of catastrophic cost. In addition, the surveys were conducted mainly in rural agrarian regions, excluding cities and pastoral regions, indicating caution for generalizability. The effect of inflation rate, which is not considered in the calculations, is another limitation that could affect the estimation.

## CONCLUSIONS

Although there was a 21% reduction between the two surveys over the 2 years, the proportion of households experiencing TB and TB-related catastrophic costs remains unacceptably high. The health sector should consider revising the policy ‘TB services exemption’, to ‘TB patient exemption’, to cover the costs of diagnosis and treatment for illnesses other than TB, including pre-diagnosis costs and diagnosis of EPTB, as well as further decentralisation of improving access to and quality of TB diagnosis and treatment services. Undertaking future studies in Ethiopia, including a national TB cost survey, by applying a stronger prospective cohort study design with all relevant determinants is essential to understand the detailed factors associated with catastrophic cost in the country.

## References

[1] World Health Organization. Tuberculosis patient cost surveys: a handbook. Geneva, Switzerland: WHO, 2021.

[2] Ghazy RM, . A systematic review and meta-analysis of the catastrophic costs incurred by tuberculosis patients. Sci Rep 2022;12(1):1–16. 35017604 10.1038/s41598-021-04345-x PMC8752613

[3] Mekonnen D, . Tuberculosis case notification rate mapping in Amhara Region-Al State, Ethiopia: Four years retrospective study. Ethiop Med J 2022;(1):3–11.

[4] Kirubi B, . Determinants of household catastrophic costs for drug sensitive tuberculosis patients in Kenya. Infect Dis Poverty 2021;10(1):1–15. 34225790 10.1186/s40249-021-00879-4 PMC8256229

[5] World Health Organization. Report 20-23. Geneva, Switzerland: WHO, 2023.

[6] Tanimura T, . Financial burden for tuberculosis patients in low- and middle-income countries: a systematic review. European Respiratory journal. 2014;43(6):1763–1775. 24525439 10.1183/09031936.00193413 PMC4040181

[7] Mullerpattan JB, . Catastrophic costs of treating drug-resistant TB patients in a tertiary care hospital in India. Indian J Tuberc. 2019;66(1):87–91. 30797290 10.1016/j.ijtb.2018.04.011

[8] Wilunda C, .; Federal Democratic Republic of Ethiopia Ministry of Health. HSTP: Health Sector Transformation Plan (2015/16–2019/20). London, UK: Ey & Ficci, 2015.

[9] Republic of Ethiopia Ministry of Health. Essential health service package. Addis Ababa, Ethiopia: MoH, 2005.

[10] World Health Organization. Global TB report, 2022. Geneva, Switzerland: WHO, 2022.

[11] Assebe LF, . Financial burden of HIV and TB among patients in Ethiopia: a cross-sectional survey. BMJ Open. 2020;10(6):e036892. 10.1136/bmjopen-2020-036892 PMC7265036 32487582

[12] Assefa DG, . Financial burden of tuberculosis diagnosis and treatment for patients in Ethiopia: a systematic review and meta-analysis. BMC Public Health. 2024;24(1):1–14. 38254019 10.1186/s12889-024-17713-9 PMC10804496

[13] Ellaban MM, . Assessment of household catastrophic total cost of tuberculosis and its determinants in Cairo: prospective cohort study. Tuberc Respir Dis (Seoul). 2022;85(2):165–174. 34814238 10.4046/trd.2021.0028 PMC8987667

[14] Republic of Ethiopia Ministry of Health. Tbl-Nsp July 2021–June 2026. Addis Ababa, Ethiopia: MoH, 2021: p 173.

[15] World Health Organization. The Global Plan to End TB 2030. Geneva, Switzerland: WHO, 2016.

[16] Central Statistical Agency Population, Ethiopia. Population projections for Ethiopia 2007–2037. Addis Ababa, Ethiopia: CSA, 2013: p 188.

[17] Aung ST, . Measuring catastrophic costs due to tuberculosis in Myanmar. Trop Med Infect Dis. 2021;6(3):983–990. 10.3390/tropicalmed6030130 PMC8293353 34287379

[18] Lu L, . Catastrophic costs of tuberculosis care in a population with internal migrants in China. BMC Health Service Res. 2020;20(1):1–9. 10.1186/s12913-020-05686-5 PMC7602335 32887605

[19] World Health Organization. Protocol for survey to determine direct and indirect costs due to TB and to estimate proportion of TB-affected households experiencing catastrophic costs due to TB. Geneva, Switzerland: WHO, 2015.

[20] Prasanna T, . Catastrophic costs of tuberculosis care: a mixed methods study from Puducherry, India. Glob Health Action. 2018;11(1):1–8. 29902134 10.1080/16549716.2018.1477493 PMC6008578

[21] Alebachew A, Mitiku W. Financing exempted health services in Ethiopia: analysis of potential policy options. Breakthrough international consultancy PLC and Harvard TH Chan School of Public Health: Addis Ababa, Ethiopia & Boston, MA, USA: Harvard TH Chan School of Public Health, 2019.

